# Active Transport, Not Device Use, Associates with Self-Reported School Week Physical Activity in Adolescents

**DOI:** 10.3390/bs9030032

**Published:** 2019-03-21

**Authors:** Ryan D. Burns, Christopher D. Pfledderer, Timothy A. Brusseau

**Affiliations:** Department of Health, Kinesiology, and Recreation, University of Utah, Salt Lake City, UT 84112, USA; chris.pfledderer@utah.edu (C.D.P.); tim.brusseau@utah.edu (T.A.B.)

**Keywords:** adolescent health, behavioral science, epidemiology, physical activity, school health

## Abstract

The purpose of this study was to examine the relationships among active transport, electronic device-use, and self-reported school week moderate-to-vigorous physical activity (MVPA) in a sample of adolescents. The sample consisted of 1445 adolescents enrolled in the Family Life, Activity, Sun, Health, and Eating study. A panel research organization invited panel members balanced to the US population on sex, census division, household income and size, and race/ethnicity. Web-based surveys were administered to each selected adolescent. Adolescents answered questions pertaining to out-of-school electronic device-use and active transport to and from school. Predicted weekly minutes of MVPA were calculated from the Youth Activity Profile. The outcome variable was predicted school week MVPA (in minutes). The predictive utility of device-use and active transport variables on self-reported school week MVPA were examined using weighted multiple linear regression models. After adjusting for age, sex, and BMI, active transport to school (b = 12.32, 95% CI [9.72–14.93], *p* < 0.001) and from school (b = 7.18, 95% CI [4.79–5.57], *p* < 0.001) were significantly associated with self-reported school week MVPA. No device-use variables were significantly associated with school week MVPA. Active transport to and from school may have an impact on school week MVPA in adolescents.

## 1. Introduction

The benefits of moderate-to-vigorous physical activity (MVPA) in adolescents span the psychomotor, affective, and cognitive domains [1,2,3]. Children and adolescents are recommended to accrue at least 60 min of MVPA per day [4]. However, as children track into adolescence, the prevalence meeting this guideline drastically decreases [5]. Fortunately, there are many opportunities throughout the day for adolescents to accrue physical activity. Active commuting to and from school, typically defined as any active mode of transport such as walking or cycling, can contribute to achieving the recommended 60 min of MVPA per day [6,7,8]. Indeed, in a recent systematic review, 81.6% of those studies examining the relationship between active transport and physical activity reported a significant positive association between active school transport and higher levels of physical activity in the pediatric population [9]. 

Although active transport may positively impact the achievement of recommended daily physical activity, barriers often prevent adolescents from participating in MVPA. One of these barriers is the excessive use of technology. Despite the fact that modern day technology, specifically electronic device use, can streamline interpersonal communication and provide various modes of education and entertainment, there is an effect on health that is concerning when used excessively within non-active contexts [10]. Because physical activity is considered a time-use behavior [11], excessive electronic device use within out-of-school settings may lead to increased sedentary behavior and decreased MVPA [12,13]. Excessive use of screen-based media is linked to lower sports and physical activity participation in children and adolescents [14]. Additionally, electronic equipment in the bedroom, consisting of televisions, phones, computers, and videogames, has been shown to negatively associate with MVPA and positively associate with BMI [15]. It has also been shown that a higher number of electronic items in the bedroom correlates with a lower estimated VO_2 Peak_ in children, an important health-related indicator [16]. In adolescents, higher video game use is associated with a poorer health status, health-related quality of life, and higher levels of depression and anxiety [17]. In teenagers, use of nearly every type of technological activity predicts indicators of poor health, relationships that are inconsistent in other pediatric populations such as preteens and younger children [18]. 

Given this past research, it is possible that excessive use of electronic devices outside of school settings may attenuate the potential benefits of active transport and perhaps reduce the probability of meeting the 60-min MVPA per day guideline. However, no study has examined these concurrent relationships among electronic device-use, active transport, and MVPA in a large sample of adolescents from the US. Fortunately, the Family Life, Activity, Sun, Health, and Eating (FLASHE) study provides useful publicly available cross-sectional data that can aid in elucidating these relationships [19]. To our knowledge, no study has considered together the relationships among electronic device-use, active transport, and school week MVPA in adolescents. Therefore, the purpose of this study was to examine the predictive relationships among active transport to and from school, electronic device-use, and self-reported school week MVPA in a sample of adolescents enrolled in the FLASHE study. 

## 2. Materials and Methods

### 2.1. Participants

Using a small effect size and an alpha level of 0.05, to achieve 80% statistical power, approximately 1484 participants would need to be recruited for multiple linear regression. Using a medium effect size, the sample needed would have to be 721 participants. A non-probability sample of adolescents from all regions of the US were recruited (N = 1445; 742 girls, 703 boys, mean age = 14.5 ± 1.6 years). Ipsos, a panel research organization, invited individuals to join its panel through print ads, internet banner ads, random digit dialing omnibus surveys, and panelist referrals [19,20]. A sample of panel members who were balanced to the US population on sex, census division, household income and size, and race/ethnicity were screened for eligibility. Within each eligible household, one adolescent (12–17 years old) and one parent were selected from the eligible residents [19,20]. The starting sample of 5027 people were invited to participate in FLASHE; of those who enrolled, 1945 were adolescents (38.7% enrollment completion rate). A total of 1479 adolescents completed all study procedures to which they were assigned (76% completion rate). Of the 1479 adolescents who completed all study procedures, 1445 recorded usable data for the current study (97.7%). Characteristics of the total sample are described elsewhere [19,20]. All subjects gave their informed consent before they participated in the study. The study was conducted in accordance to the Declaration of Helsinki. This study received approval from the Office of Management and Budget (#0925-0686; 12/13/13), the NIH Institutional Review Board (#327123; 05/30/13), and the Westat Institutional Review Board (#6053.01.01; 03/14/13) [19,20]. Written informed consent was obtained from the adolescents prior to data collection.

### 2.2. Procedures

Web-based surveys were administered to each adolescent (aged 12–17-years-old) between April to October of 2014 [19,20]. Analysis weights were used to reduce sampling bias within person-level analysis [19,20]. The dependent variable was self-reported school week adolescent MVPA (in minutes). Adolescent weekly MVPA (in minutes) was calculated using the validated Youth Activity Profile (YAP; [predicted in-school minutes + predicted out-of-school minutes]—predicted weekend minutes) [21], which was incorporated within the FLASHE adolescent Physical Activity survey [19,20,21]. It has been shown that the YAP offers good utility for large-scale research to characterize physical activity and sedentary behavior in adolescents [21].

### 2.3. Data Processing

There were two sets of predictor variables. The first set of predictor variables consisted of items representing time-use on phones, computers, video games, and television. The device-use items were scored on a 1–5 Likert-type scale (1 = “less than 1 h per day”, 5 = “more than 3 h per day”) and asked “How much time did you spend watching TV outside of school time? This includes time spent watching movies or sports but not time spent playing video games.” For television-use, “How much time did you spend playing video games outside of school time? This includes games on Nintendo DS, Wii, Xbox, PlayStation, iTouch, iPad, or games on your phone.” For video game use, “How much time did you spend using computers outside of school time? This doesn’t include homework time but includes time on Facebook as well as time spent surfing the internet, instant messaging, playing online video games or computer games.” For computer use and phone use, “How much time did you spend using your cell phone after school? This includes time spent talking or texting.” 

The second set of predictor variables consisted of items representing active transport to and from school. The active transport items were scored on a recoded 1–5 Likert-type scale (1 = “0 days (never)”, 5 = “4–5 days”) and asked “How many days did you walk or bike to school? If you can’t remember, try to estimate.” and “How many days did you walk or bike from school? If you can’t remember, try to estimate.” for the “to” and “from” school active transport items, respectively. 

### 2.4. Statistical Analysis

Data were screened for outliers using boxplots and z-scores and checked for Gaussian distributions using k-density plots. Differences between sexes on all observed variables were examined using independent t-tests. Effect sizes were calculated using Cohen’s delta (d), where d < 0.20 indicated a small effect size, d = 0.50 indicated a medium effect size, and d > 0.80 indicated a large effect size [22]. The predictive utility of device-use and active transport variables on adolescents’ self-reported school week MVPA were examined using weighted multiple linear regression models. Predictors were entered into the model using block-wise entry. The first block (Model 1) consisted of the device-use predictor variables. The second block (Model 2) consisted of the predictors of Model 1 plus the two active transport variables. The third entry block (Model 3) consisted of the predictors of Model 2 plus the potential confounders of age, sex, and BMI. Effect modification was also tested by creating interaction terms combining each predictor with age or sex. To ensure no multicollinearity was present among predictors, variance inflation factor (VIF) scores were computed, with a VIF > 5.0 indicating the presence of multicollinearity. Reporting of the results consisted of communicating the unadjusted and adjusted b-coefficients with 95% confidence intervals. For predictors yielding noteworthy significant effects, marginal predicted effects were graphically displayed. All analyses had an initial alpha level of *p* < 0.05 and were carried out using the STATA v15.0 statistical software package (College Station, TX, USA). 

## 3. Results

Descriptive statistics are presented in Table 1. Girls reported higher phone use (mean difference = 0.60, *p* < 0.001, d = 0.43) compared to boys. Boys reported higher video game use (mean difference = 0.90, *p* < 0.001, d = 0.75), higher active transport to school (mean difference = 0.29, *p* < 0.001, d = 0.19), and higher active transport from school (mean difference = 0.15, *p* < 0.001, d = 0.09) compared to girls. There were no differences between sexes in school week minutes of MVPA. 

**Figure 1:**
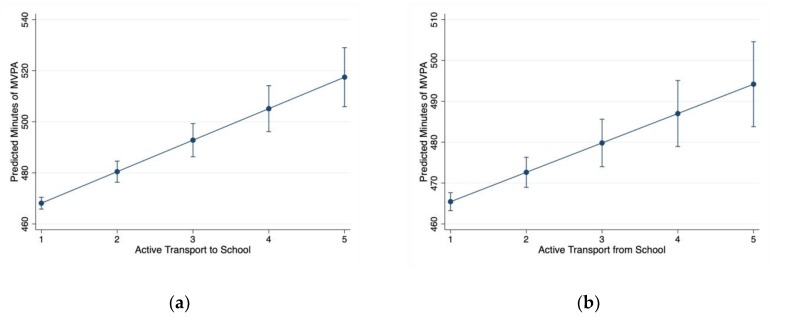
Marginal predicted effects on self-reported school week moderate-to-vigorous physical activity as a function of self-reported active transport to and from school. (**a**) Relationship between active transport to school and predicted weekly minutes of MVPA; (**b**) Relationship between active transport from school and predicted weekly minutes of MVPA. Note: bars are 95% confidence intervals.

Table 2 presents the main effect parameter estimates from the weighted multiple linear regression models. The highest VIF observed was 2.95 for the active transport to school predictor; therefore, it was assumed that multicollinearity was not present within any of the models. For Model 1, most device-use predictors, except for television use (*p* = 0.664), were significantly associated with school week MVPA (*p* < 0.05). Two predictors in Model 1 (phone and computer-use) showed a negative association with MVPA (i.e., higher use relating to lower MVPA) and one predictor (video game use) interestingly showed a positive association with MVPA (higher use relating to higher MVPA). In Model 2, the same patterns of association were observed as in Model 1 with the addition of two active transport predictors, which both showed statistically significant positive associations with MVPA (*p* < 0.001). For Model 3, only active transport predictors and the age and sex covariates were significantly associated with MVPA (*p* < 0.01). Specifically, higher levels of school active transport was associated with higher levels of school week MVPA, being female was associated with lower levels of school week MVPA, and older age was associated with lower levels of school week MVPA. Within Model 3, there were no statistically significant interactions with age or sex. Figure 1 shows the predicted marginal effects on school week MVPA for active transport to and from school.

## 4. Discussion

The purpose of this study was to examine the relationships among out-of-school electronic device use, active transport to and from school, and self-reported MVPA in a sample of adolescents enrolled in the FLASHE study. The results indicated that active transport to and from school were significantly associated with an adolescent meeting the 60 min of MVPA per day guideline. After considering active transport, age, sex, and BMI, no device-use variables were significantly associated with school week MVPA. To the authors’ knowledge, this is the first study examining the concurrent relationships among these variables in a large sample of US adolescents. Information gained from this study can be used to devise interventions targeting active transport in the adolescent population. Discussion of the results and practical implications for public health practice are discussed further. 

The salient finding from this study was that active transport to and from school significantly related to school week MVPA. These relationships were independent of electronic device use, age, sex, and BMI. These findings disagree with some past cross-sectional studies examining the relationship between active transport to school and MVPA [23]. Reasons for discordance in results may be geographical (e.g., country location) and the assessment of MVPA (e.g., accelerometry vs. self-report). Nonetheless, sedentary behavior and MVPA are both time-use behavioral variables; therefore, increases in sedentary behaviors inherently decrease physical activity (i.e., light physical activity and MVPA) [24]. Sedentary behavior, unfortunately, also relates to poorer cognitive functioning and classroom behavior in children, adolescents, and adults [25,26]. Decreasing sedentary behavior and increasing MVPA have been important public health messages for decades. One of the ways to decrease sedentary behavior and increase MVPA is via active transport. As stated previously, many studies have found relationships with children engaging in active transport and higher levels of daily physical activity [9]; however, this study supports that this relationship is independent of electronic device use. 

Incentivizing active transport among adolescents remains a problem as past research has identified several barriers. Observed barriers to active transport include traffic, crime, danger, long distance, weather [27], a lack of friends that live in the neighborhood [28], poor neighborhood accessibility [29], not having enough time [30], negative attitudes toward active commuting [31], low-visibility, and the absence of parks [32]. Indeed, although the average distance from home to school has increased in the past few decades [33], it has been shown that almost half of children living within 1 mile of their school still passively commute by car [34]. Finding ways to remove or decrease the impact of these real and/or perceived barriers is an important line of research in the public health field, especially in lower income adolescents where the built environment and issues with crime may preclude active transport to and from school and especially as adolescents track into adulthood where the prevalence of active transport significantly decreases [35]. 

Another important finding from this study was that out-of-school electronic device use did not significantly relate to school week MVPA. Although most relationships between electronic device use and physical activity were significant in Model 1, after taking into consideration active transport and the potential confounders of age, sex, and BMI, the computer, phone, and video game effects became non-significant (see Model 3). A confounding variable that may have affected these relationships is the location of screen-based media use and the portability of the electronic device. An area of the home that represents a particularly important and frequent location of screen-based media use among adolescents is the bedroom. Studies have shown that access to screens and other electronic media specifically in the bedroom, is associated with a number of detrimental effects, including higher sedentary time and screen time [36,37], lower reading time and higher BMI, waist circumference, and body fat [38], less time spent in vigorous physical activity, lower fruit and vegetable intake, and a lower grade point average [39]. Electronic device use in other locations may be more transient. Additionally, devices used outside of the bedroom may be more portable. Portable devices may not preclude physical activity participation, and may in fact promote physical activity via various fitness-based applications [40]. Therefore, the construct of electronic device use, as examined in the current study, may be too broad because device location and device portability may be important characteristics when considering the relationships with physical activity [41]. These relationships should be examined with additional research. 

Public health messages should continue to communicate the importance of improving physical activity via active transport to and from school in the adolescent population. Given the results of this study, limiting the use of electronic devices may need greater specificity, possibly depending on the location and the portability of the electronic device. A positive aspect to the findings was the robustness of the active transport variables in their relationship with school week MVPA. Active transport is a valuable method to be used to improve the health behaviors of adolescents. Health educators, physical educators, and public health professionals working with adolescents to improve health should recommend active transport as a valuable means to improve daily physical activity levels, especially in lower income populations where the built environment provides additional barriers [42]. 

There are limitations to this study that must be considered before the results can be generalized. First, a non-probability convenience sample of adolescents were recruited, which limits external validity. Second, MVPA was assessed by means of self-report; therefore, recall and social-desirability biases may attenuate internal validity. Future research should examine the relationships using more objective assessments of sedentary behavior and physical activity, such as accelerometry, given the unknown reliability and validity of self-report measures in specific populations of youth [43]. Third, specific means of active transport (e.g., walking, biking, scooter, etc.) were not assessed and accounted for in the analysis, nor was the use of semi-active transport where both active and public transport are used (e.g., taking the school bus and walking/biking to school). Specific modes of active transport may have confounded the results due to a variation in ambulatory movement and the use of semi-active transport may have provided additional useful information regarding specific patterns of behavior. Fourth, geographical location, environmental factors (e.g., walking paths, green space, safety, etc.), and weather patterns, all of which affect active transport, were not accounted for and may have confounded the results as adolescents within less supportive built environments and unfavorable psychosocial variables tend to not to utilize active transport [44]. Finally, only age and sex were tested for effect modification. Other potential effect modifiers that could be examined in future research may include socioeconomic status, after-school sports participation, physical maturation, and salient psychosocial variables such as physical activity enjoyment and self-efficacy. 

## 5. Conclusions

In conclusion, active transport significantly associates with school week MVPA. After accounting for active transport and potential confounding variables, no device-use variables significantly related to school week MVPA. In order to ensure that adolescents meet physical activity standards, public health messages should continue to emphasize the benefits of active transport to and from school. Even though electronic device use may contribute to sedentary behavior in the home environment, active transport may be a more important factor to consider given the results from this study. Future research should explore these relationships using greater specificity of electronic device use, such as location of use and portability (e.g., portable vs. non-portable). This study provides additional evidence of the importance of active transport to improve the health behaviors of adolescents and suggests that engaging in active transport may have stronger links with habitual weekly physical activity compared to out-of-school electronic device use in the adolescent population.

## Figures and Tables

**Table 1:** Descriptive statistics for the total sample and within sex groups (means and standard deviations).

	Total Sample (N = 1445)	Girls (n = 742)	Boys (n = 703)
Age (years)	14.5 (1.6)	14.4 (1.6)	14.5 (1.6)
BMI (kg/m^2^)	22.2 (4.9)	22.2 (5.0)	22.2 (4.7)
Phone Use	2.7 (1.4)	**3.0 ^†^ (1.4)**	2.4 (1.3)
Video Game Use	2.6 (1.3)	2.1 (1.2)	**3.0 ^†^ (1.2)**
Computer Use	2.8 (1.3)	2.8 (1.3)	2.8 (1.2)
Television Use	3.2 (1.1)	3.2 (1.1)	3.2 (1.2)
AT to School	1.68 (1.4)	1.55 (1.29)	**1.84 ^†^ (1.50)**
AT from School	1.80 (1.4)	1.72 (1.42)	**1.87 ^†^ (1.53)**
Weekly MVPA (min)	468.3 (97.4)	466.3 (95.9)	470.6 (99.1)

BMI stands for body mass index; AT stands for active transport; MVPA stands for moderate-to-vigorous physical activity; device use and active transport were scored on a 1–5 Likert scale; bold and † denote statistical differences between sexes, *p* < 0.05.

**Table 2:** Parameter estimates from the weighted multiple linear regression models.

	Model 1 b-coefficient (95% CI)	Model 2 b-coefficient (95% CI)	Model 3 b-coefficient (95% CI)
Phone use	**−11.81 ^†^ (−15.79–−7.83)**	**−12.81 ^†^ (−16.55–−9.06)**	1.23 (−0.48–3.96)
Video Game Use	**8.99 ^†^ (4.78–13.20)**	**7.80 ^†^ (3.82–11.77)**	−0.22 (−2.03–1.60)
Computer Use	**−8.00 ^†^ (−12.35–−3.65)**	**−8.20 ^†^ (−12.30–−4.10)**	1.12 (−0.69–2.93)
Television Use	0.76 (−3.98–5.51)	0.86 (−3.65–5.36)	0.19 (−1.77–2.16)
AT to School		**14.30 ^†^ (7.56–21.03)**	**12.32 ^†^ (9.72–14.93)**
AT from School		**10.27 ^†^ (4.07–16.46)**	**7.18 ^†^ (4.79–9.57)**
Age (years)			**−52.8 ^†^ (−54.4–−51.3)**
Sex (boy referent)			**−7.5 ^†^ (−12.3–−2.7)**
BMI (kg/m^2^)			−0.33 (−0.76–0.09)

Outcome is weekly moderate-to-vigorous physical activity (minutes); Model 1 = device use predictors; Model 2 = Model 1 + active transport predictors; Model 3 = Model 2 + potential confounding variables; 95% CI stands for 95% confidence interval; AT stands for active transport; BMI stands for body mass index; device use and active transport were scored on a 1–5 Likert scale; bold and † denote statistical significance, *p* < 0.05.
